# Simple Flow-Based System with an In-Line Membrane Gas–Liquid Separation Unit and a Contactless Conductivity Detector for the Direct Determination of Sulfite in Clear and Turbid Food Samples

**DOI:** 10.3390/membranes10050104

**Published:** 2020-05-18

**Authors:** Aulia Ayuning Tyas, Thitaporn Sonsa-ard, Kanchana Uraisin, Duangjai Nacapricha, Phoonthawee Saetear

**Affiliations:** Flow-Innovation Research for Science and Technology Laboratories (FIRST Labs), Department of Chemistry and Center of Excellence for Innovation in Chemistry, Faculty of Science, Mahidol University, Bangkok 10400, Thailand; auliaaa22@gmail.com (A.A.T.); t.sonsa.ard@gmail.com (T.S.-a.); u_kanchana@hotmail.com (K.U.); dnacapricha@gmail.com (D.N.)

**Keywords:** sulfite, turbid sample, turbidity, contactless conductivity, gas diffusion

## Abstract

This study presents a simple flow-based system for the determination of the preservative agent sulfite in food and beverages. The standard method of conversion of sulfite ions into SO_2_ gas by acidification is employed to separate the sulfite from sample matrices. The sample is aspirated into a donor stream of sulfuric acid. A membrane gas–liquid separation unit, also called a ‘gas-diffusion (GD)’ unit, incorporating a polytetrafluoroethylene (PTFE) hydrophobic membrane allows the generated gas to diffuse into a stream of deionized water in the acceptor line. The dissolution of the SO_2_ gas leads to a change in the conductivity of water which is monitored by an in-line capacitively coupled contactless conductivity detector (C4D). The conductivity change is proportional to the concentration of sulfite in the sample. In this work, both clear (wine) and turbid (fruit juice and extracts of dried fruit) were selected to demonstrate the versatility of the developed method. The method can tolerate turbidity up to 60 Nephelometric Turbidity Units (NTUs). The linear range is 5–25 mg L^−1^ SO_3_^2−^ with precision <2% RSD. The flow system employs a peristaltic pump for propelling all liquid lines. Quantitative results of sulfite were statistically comparable to those obtained from iodimetric titration for the wine samples.

## 1. Introduction

Sulfite is usually added in various forms to preserve food and beverages. Sulfite has the ability to inhibit bacterial growth and chemical processes by either enzymatic or non-enzymatic reaction [1,2]. The active species is free sulfite. However, it is difficult to determine free sulfite due to its low stability. The determination of total sulfite, which is the sum of free sulfite and bound sulfite, is more reliable than only the free sulfite. Release of bound sulfite from other molecules can be carried out by heating or adding alkaline media [3]. Sulfite can cause adverse symptoms such as asthmatic, gastrointestinal distress, diarrhea and hives for certain people [4,5]. According to Joint FAO/WHO Expert Committee on Food Additives (JECFA), the acceptable daily intake of sulfite is 0.7 mg kg^-1^ body weight per day [6]. The United States Food and Drug Administration (FDA) has announced that all food and beverage products containing sulfite must be labelled on the package as “contains sulfites”, if sulfite content is more than 10 mg L^−1^ SO_3_^2−^ [7]. Hence, to protect customer’s health and safety, monitoring of total sulfite in food and beverage products is essential. 

An optimized Monier-Williams method is one of the standard methods recommended by the Association of Analytical Chemist (AOAC) (Official Method 990.28). This method employs a distillation process to liberate SO_2_ gas from the sample solution containing hydrochloric acid. Purging nitrogen gas during distillation process is necessary to assist trapping of the produced SO_2_ gas in the hydrogen peroxide solution, leading to formation of sulfuric acid. The solution is then titrated with standard sodium hydroxide. Another titrimetric method is the Ripper method [8]. The sample solution is also acidified in order to convert sulfite to SO_2_ gas. The SO_2_ gas is then titrated with standard iodine using starch indicator to obtain the amount of sulfite in the sample solution. These two titrimetric methods are based on separation of sulfite from the sample matrices by acidification according to the following reaction [9]:

SO_3_^2−^ + 2H_3_O^+^ ⇌ SO_2_ + 3H_2_O (1)

Nevertheless, these methods are laborious, time consuming, and have low precision because of loss of SO_2_ gas due to operating in an open system.

The reaction shown in Equation (1) can be applied for converting sulfite into SO_2_ gas in food and beverages containing complex matrices. There is an available commercial analyzer for total sulfite and SO_2_/H_2_S from UIC, Inc., IL, USA [10]. This analyzer is based on coulometric detection of SO_2_ gas after acidification of sample. Analysis time is 5 to 7 min and is suitable for both solid and liquid samples. 

One of the common gas–liquid separation methods is gas-diffusion (GD) through a membrane. The general configuration of a GD unit consists of two acrylic blocks with designed grooves. A hydrophobic membrane, e.g., polytetrafluoroethylene (PTFE), is placed between the two acrylic blocks to allow only hydrophobic gas to pass. Hydrophobic PTFE membrane can improve the selectivity of the analysis by allowing rapid rate of gas diffusion with good chemical resistance. In sulfite analysis, after acidification, the generated SO_2_ gas in the donor stream passes through the hydrophobic membrane and dissolves in the acceptor stream [11,12]. Several detection methods for flow-based systems have been reported for the detection of the dissolved SO_2_, such as pH-ion-sensitive field-effect transistor (pH-ISFET) [13], colorimetry [14,15,16], chemiluminescence [17], potentiometry [18], voltammetry [19], amperometry [20,21], biamperometry [9], and conductivity [11]. 

Another configuration of the gas–liquid separation unit is the membraneless vaporization (MBL-VP) unit. A unit that is suitable for computer control of flow was first proposed by Ratanawimarnwong et al. in 2013 [22]. The unit has a headspace region connecting the donor and acceptor chambers. The flows in and out of the cones are through ports at the base of the cones. A capacitively coupled contactless conductivity detector (C4D) has been used as a detection system for the MBL-VP unit. C4D is a universal detector for conductivity measurements but with the two electrodes not directly contacting the measuring solution. C4D has advantages over conventional conductivity detector for flow systems since the electrodes are easily mounted on the outer wall of the flow tube and is robust in use. Several applications of MBL-VP-C4D for the analysis of volatile gases converted from ionic analytes have been presented, such as dissolved ammonium and sulfide in canal water [23] and sulfite in wines [24]. 

In this work, C4D was selected as the detector for the analysis of total sulfite after the conversion of the sulfite ions into SO_2_ gas via acidification. The samples are wines, extracts of dried fruit and turbid fruit juice. MBL-VP unit with C4D has been reported for analysis of sulfite in clear samples, especially wine, but there is no report of analysis of turbid or cloudy sample such as extracts of dried fruit or fruit juice. Particulate suspensions can cause the clogging of injection valves and narrow flow channels in the MBL-VP unit. We therefore selected the GD unit with hydrophobic PTFE membrane for gas–liquid separation. Direct aspiration for a fixed time of diluted turbid sample into the acid donor stream is employed. The generated SO_2_ gas from the donor stream passes through the PTFE membrane in the GD unit and dissolves in the water acceptor leading to a change in the conductivity of the water. This conductivity change is monitored by the C4D detector.

## 2. Materials and Methods 

### 2.1. Chemicals and Preparation of Sulfite Standard Solutions

All chemicals and reagents used were analytical reagent grade. All solutions were prepared in a deionized (DI) Milli-Q^®^ Advantage A10 Water Purification System (resistivity 18.2 MΩ·cm, Millipore SAS, Molsheim, France). Stock standard sulfite of 1000 mg L^−1^ SO_3_^2−^ was freshly prepared by dissolving 0.1575 g of Na_2_SO_3_ (Merck, Darmstadt, Germany) in 100.0 mL of 0.1% (w/v) Na_2_EDTA (Fisher scientific, Loughborough, UK). The accurate concentration of this stock standard solution was determined by titration with standardized iodine solution. A stock solution of 20% (w/w) sugar (food grade from Mitr Phol Sugar, Thailand) was prepared by weighing exactly 10.00 g of table sugar followed by the addition of 40.00 g of deionized water.

A working sulfite standard for the determination of total sulfite was freshly prepared from the 1000 mg L^−1^ SO_3_^2−^ stock solution by aliquoting appropriate volumes to give a series of sulfite standards (5 to 25 mg L^−1^ SO_3_^2−^). For the analysis of wine samples, to each aliquot of the stock sulfite solution, 0.50 mL of 5% (w/v) Na_2_EDTA and 2.50 mL of 4 mol L^−1^ NaOH (Merck, Germany) were added and then the solution made up to volume with DI water in a 25.00-mL volumetric flask. For the analysis of dried fruit extracts and turbid fruit juices, 2.50 mL of 20% (w/w) sugar was also added into each aliquot of standard sulfite solution. A working sulfite standard for the determination of free sulfite was freshly prepared in the same manner as for the determination of total sulfite, but without the addition of the NaOH solution. 

### 2.2. Preparation of Samples

White and red wines, turbid fruit juice and various packaged dried fruits were purchased from local supermarkets in Bangkok. 

For the analysis of total sulfite in wine and fruit juice, 2.50 mL of wine/juice was aliquoted into a 25.00-mL volumetric flask. Then, 0.50 mL of 5% (w/v) Na_2_EDTA and 2.50 mL of 4 mol L^−1^ NaOH were added. Deionized water was added to make up to volume.

**Figure 1:**
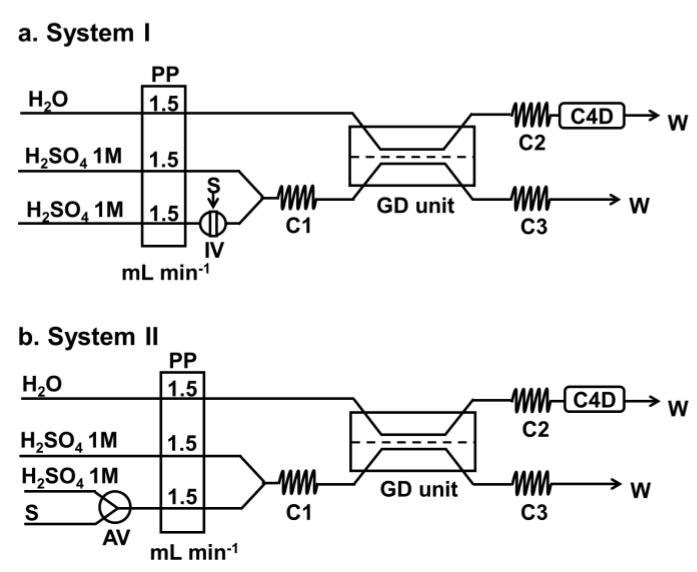
Schematic of the flow-injection systems with an in-line GD unit and a C4D detector. System I with injection valve (IV) is used for clear samples and for the optimization study. System II allows for aspiration of sample via the 3-way valve (AV) and is used for both clear and turbid samples. PP: peristaltic pump; IV: injection valve; AV: 3-way aquarium valve; C1 and C2: mixing coils; C3: back-pressure coil; GD: gas diffusion unit; C4D: capacitively coupled contactless conductivity detector; S: sample; W: waste.

For the analysis of total sulfite in dried fruit, an extraction step, adapted from Jiangli Lin et al. [25] was employed. The dried fruit sample was manually cut into small pieces and then ground in a blender. A 40.00-mL volume of 0.4 M NaOH in 0.1% (w/v) Na_2_EDTA was added into a 50-mL centrifuge tube containing 8.00 g of the blended dried fruit. The mixture was sonicated for 15 min. The sample was then directly introduced via aspiration into the donor flow line (see Figure 1b).

### 2.3. Flow Systems and Operation

Figure 1 shows the two flow systems used in this work for analysis of the two types of samples, i.e., clear liquid and turbid samples. System I is the flow-injection analysis (FIA) system suitable for clear samples and was employed for optimizing various flow parameters (see Section 3). System II is the flow system employed for the final developed method for analysis of turbid and clear samples. Both systems are coupled with an in-line gas-diffusion (GD) unit and a capacitively coupled contactless conductivity detector (C4D).

In both flow systems, a peristaltic pump (Ismatec model ISM 827, Glattbrugg-Zürich, Switzerland) is employed to propel the donor and acceptor solutions. The donor stream is sulfuric acid (1 mol L^−1^) and the acceptor is deionized water. In order to introduce the sample into the flow line, an injection valve (Rheodyne^®^ Model 5041, Cotati, CA, USA) mounted with a 200-µL sample loop is used in flow System I. In flow System II a 3-way aquarium flow valve (Pawfly-UL232, New York, NY, USA) is used to aspirate a sample solution at a flow rate of 1.5 ml min^-1^ for 12 s into the donor flow by opening the valve. The pump tubing is Tygon^TM^ (Cole-Parmer, Vernon Hills, IL, USA), with polytetrafluoroethylene (PTFE) tubing (i.d. 1.02 mm) for the liquid-flow path and for the mixing coils C1–C3. 

**Figure A1:**
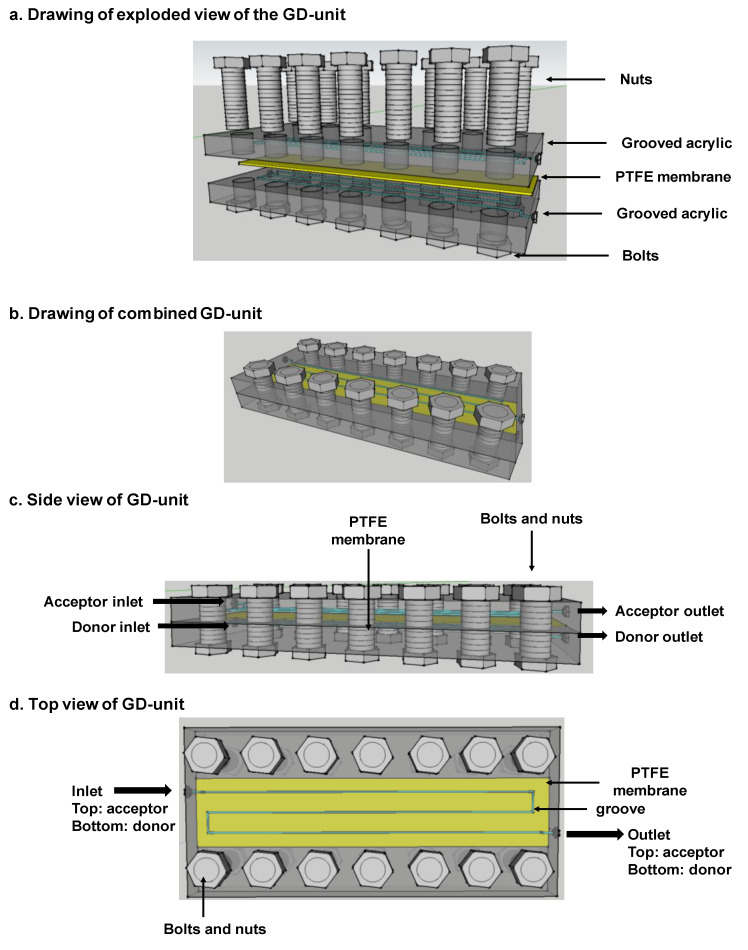
Drawing of gas-diffusion unit used in this work.

The rectangular GD unit (5.0-cm width, 15.0-cm length, 2.0-cm height) is made from two rectangular pieces of clear Perspex blocks with matching grooves comprising 3 parallel straight tracks (1-mm depth) with each end the middle track connected at right angles to the two outer tracks as shown in Figure A1 (see Appendix A in supplementary data). Each groove is connected to an inlet and outlet port. A polytetrafluoroethylene (PTFE) plumber tape (0.1-mm thick and 16-mm wide) is placed between the two Perspex blocks, thus producing channels above and below the membrane. The unit is made leak tight by 14 pairs of nuts and bolts. 

The C4D detection flow cell [23,24,26] is a polyether ether ketone (PEEK) tubing (1.0 mm i.d., 1.6 mm o.d.) with two cylindrical bands of painted silver conductive ink (2.0 cm long and 0.5 mm apart) as electrodes. Shielding is carried out by placing the PEEK tubing in a metallic box. An AC voltage (20 V_pp_, 20 kHz) is applied to one electrode from a function generator (GW Instek, SFG-2104, Taiwan). The AC current flowing between the two electrodes is monitored at the second electrode, The AC current is amplified and rectified by a custom build electronics unit (Bangkok High Lab Co., Ltd., Bangkok, Thailand). The output DC signal, which is proportional to the circuit admittance, is recorded by a signal recorder (e-corder 201, eDAQ, Denistone East, NSW, Australia) and analyzed with eDAQ Chart software (version 5.5.25).

**Figure 2:**
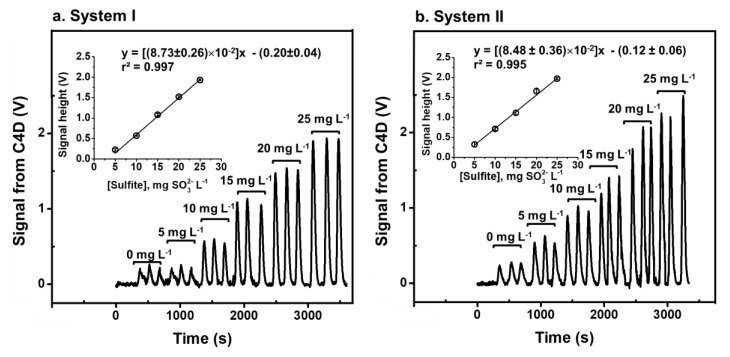
Signal profiles of standard sulfite solutions obtained from **(a)** System I and **(b)** System II. Insets are the linear calibration graphs after the subtraction of reagent blank signal (0 mg SO_3_^2−^ L^−1^).

**Figure A2:**
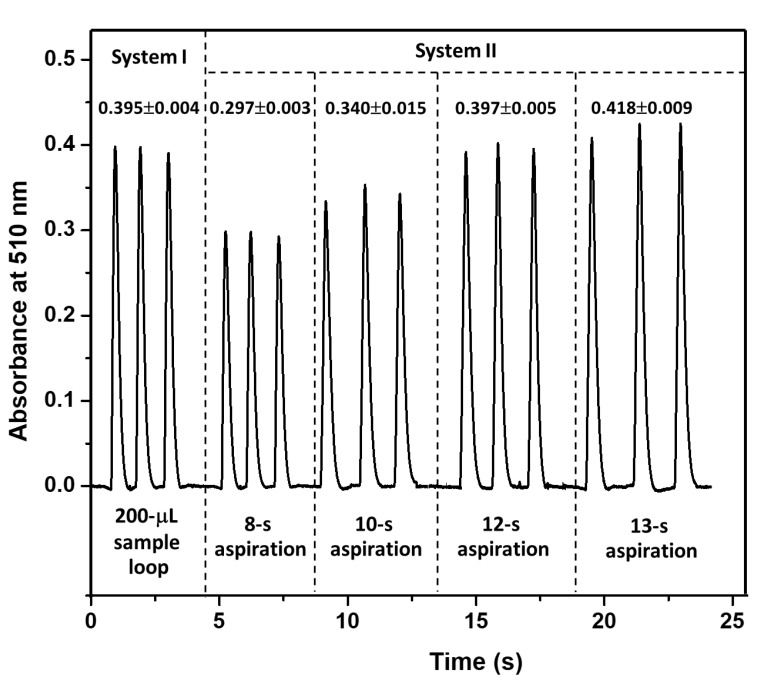
Comparison of absorbance signals of red dye solution obtained from System I using the 200-μL sample loop and from System II with various aspiration times (8–13 s) for sample introduction.

In System I (Figure 1a), standard/sample is introduced via a sample injection loop, whereas, in System II, the standard/sample is aspirated into the flow system (as described above) to react with a continuous flowing stream of sulfuric acid. Aspiration time for sample introduction in System II was studied as shown in Figure A2 (see Appendix B in data). The sulfite ion is converted to SO_2_ gas, which diffuses across the PTFE membrane to dissolve into the water acceptor stream. The C4D flow cell then monitors the signal change resulting from conductivity change of water upon the dissolution of the SO_2_ gas. Calibration graph is a plot of the C4D signal heights against the concentrations of the injected standard, as shown in the insets in Figure 2.

A new PTFE membrane is employed for every new set of measurements. During an analysis, a control standard solution of 10 mg L^−1^ SO_3_^2−^ is analyzed after every six samples to assess the condition of the PTFE membrane based on whether the concentration of the control is within ±3SD, standard deviation, of the back-calculated concentration using the calibration equation. 

## 3. Results and Discussion

### 3.1. Flow System Design

In a preliminary study to investigate the potential use of the flow injection system with the GD unit for determination of sulfite, standard sulfite solutions were directly injected via an injection valve (IV in Figure 1a) into a continuous flowing reagent stream of 1.0 M H_2_SO_4_ (cf. Figure 1a without the middle flow line). However, it was found that the C4D signals were not reproducible (data not shown). Thus, a second flow line of 1.0 M H_2_SO_4_ was added to provide better mixing after generation of SO_2_ gas (see Figure 1a). The signal profiles were found to be reproducible, as shown in Figure 2a. A good linear calibration range was also obtained from 5 to 25 mg L^−1^ SO_3_^2−^ (see inset of Figure 2a).

To overcome the problem of clogging of the injection valve (IV in Figure 1a) by turbid samples, even after sample dilution, flow System II (Figure 1b) was designed for aspiration of the sample directly into the flow line. A 3-way aquarium flow valve (AV) was employed for the introduction of such turbid samples. When the valve is set to connect to the standard sulfite/sample reservoir for 12 s, the solution is aspirated into the donor flow line (flow rate 1.5 mL min^−1^). The flow System II provides comparable sensitivity for the same linear range of 5–25 mg L^−1^ SO_3_^2−^ and coefficient of determination (*r*^2^) as for System I (see insets in Figure 2a,b). To confirm that the slopes of calibrations are not significantly different, a paired *t*-test of the back-calculated concentrations using the calibration equations was performed with *t*_stat_ (0.68) < *t*_crit_ (3.16) at *p* = 0.05 (see Table A1, Appendix C in supplementary data). In this study, System II was selected as the suitable system for the determination of total sulfite of both clear and turbid samples.

### 3.2. Optimization

In the optimization study, System I was employed for convenience.

#### 3.2.1. Concentration of the Sulfuric Acid

**Figure 3:**
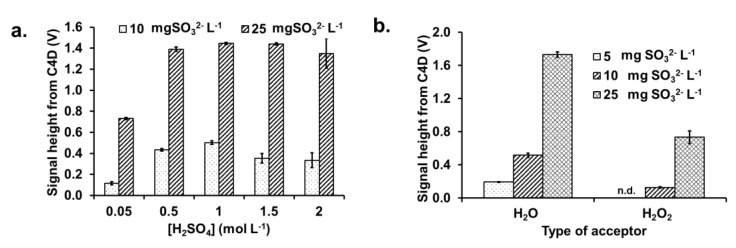
Bar graphs of the effect of (**a**) sulfuric acid concentration in the donor stream and (**b**) the type of acceptor solution on C4D signal heights. n.d.: not detected.

The concentration of H_2_SO_4_ in the donor stream affects the extent of the conversion of the sulfite ion into SO_2_ gas. The concentration range of H_2_SO_4_ studied was 0.05 to 2.0 mol L^−1^, using sulfite standard solutions of 10 and 25 mg L^−1^ SO_3_^2−^, respectively. The flow rates for the flow lines are shown in Figure 1a. As shown in Figure 3a, it is observed that the C4D signal heights are small when 0.05 mol L^−1^ H_2_SO_4_ is used (~0.11 V and ~0.73 V, for 10 and 25 mg L^−1^ SO_3_^2−^, respectively). The C4D signal heights significantly increased fourfold for the 10 mg L^−1^ SO_3_^2−^ sample and twofold for 25 mgSO_3_^2−^ L^−1^ when the H_2_SO_4_ concentration is increased to 0.5 mol L^−1^, with the heights remaining constant for higher concentrations of the acid. It should be noted that, for flow systems, the chemical processes have not reached equilibrium. These results show that, at ≥0.5 mol L^−1^, sufficient excess of acid has been achieved for giving a constant signal (see Figure 3a). Thus 1.0 mol L^−1^ H_2_SO_4_ was selected to ensure that the C4D signal was independent of the acid concentration.

#### 3.2.2. Selection of Type of Acceptor Liquid

The acceptor stream should be a solution that gives a change of conductivity on the dissolution of the diffused SO_2_ gas. In this study, two acceptor liquids were tested, i.e., DI water and 1mM H_2_O_2_ [11]. Figure 3b shows the signals from the C4D obtained from sulfite standards (5, 10 and 25 mg L^−1^ SO_3_^2−^) using the two acceptor liquids. The results in Figure 3b clearly show that only deionized water gave significant changes in conductivity for all three sulfite standard solutions (5, 10 and 25 mg L^−1^ SO_3_^2−^). When employing 1mM H_2_O_2_ as acceptor liquid, changes in the C4D signals were found for only sulfite at 10 and 25 mg SO_3_^2−^ L^−1^, respectively. When SO_2_ gas dissolves in the acceptor liquid, the conductive species produced in water and H_2_O_2_ are H_3_O^+^ and SO_3_^2−^ and H_3_O^+^ and SO_4_^2−^, respectively. Both deionized water and H_2_O_2_ have no conductivity because they have low dissociation constants (water: pK_a_ 7 and H_2_O_2_: pK_a_ 11.75). DI water was selected because it is environmentally friendly and provides a lower limit of detection.

#### 3.2.3. Flow Rate of Donor and Acceptor Streams

The flow rates of donor and acceptor streams were investigated to obtain highest sensitivity. The flow rate of donor was varied whilst keeping the acceptor flow rate constant and vice versa. 

**Figure 4:**
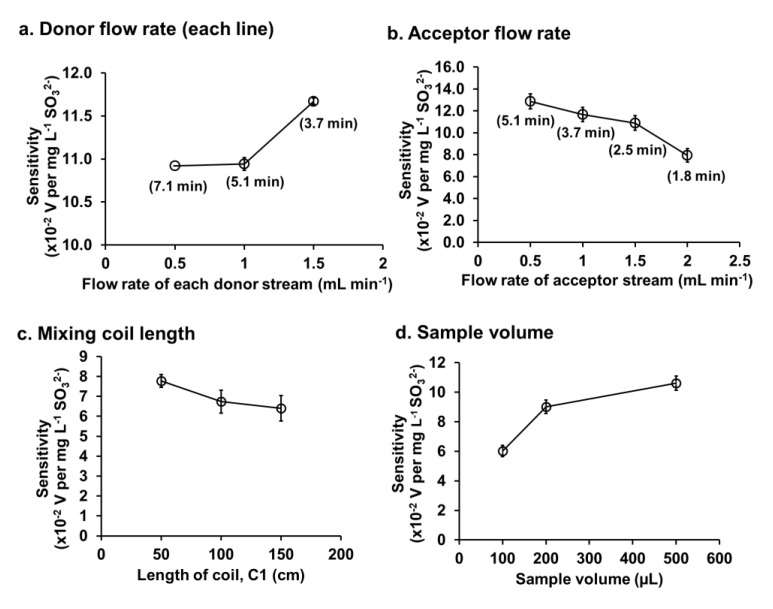
Optimization of physical parameters affecting the sensitivities and analysis time for sulfite analysis. The numbers in parenthesis are analysis time (min) per injection.

The flow rate of each donor streams was varied from 0.5 to 1.5 mL min^−1^, with the flow rate of acceptor line fixed at 1.0 mL min^−1^. Figure 4a shows that increasing the flow rate of donor stream gave an increase in the sensitivity. Moreover, higher flow rate also reduced the analysis time (see the numbers in parenthesis in Figure 4a). Therefore, the flow rate of 1.5 mL min^−1^ was selected as the optimum flow rate of each donor line.

Figure 4b shows the effect of flow rate of the acceptor on sensitivity, with each donor flow line set at the selected value of 1.5 mL min^−1^. It was found that increasing the flow rate of the acceptor from 0.5 to 2.0 mL min^−1^ produced a large decrease in the sensitivity (from 12.9 to 7.9 (×10^−2^) V per mg L^−1^ SO_3_^2−^). High flow rate gives a short residence time of the acceptor solution in the gas diffusion unit and thus a lower accumulated concentration of the SO_2_ gas, i.e., a lower C4D peak height. However, the flow rate of the acceptor stream of 1.5 mL min^−1^ was selected as a compromise between sensitivity and analysis time. 

#### 3.2.4. Length of Mixing Coil C1

Mixing coils in flow Systems I and II (Figure 1) are employed for different purposes. Coil C1 is used to improve the mixing of the sample and the acid donor stream. Coil C2 is also used to provide efficient mixing of the dissolved SO_2_ gas in the water acceptor. Coil C3 is employed to produce back pressure in the donor line inside the GD unit. In this work, we need to minimize the dispersion effect in the acceptor stream so coil C2 is kept as short as possible at 50 cm. In this experiment, only coil C1 was varied from 50 to 150 cm. As shown in Figure 4c, the 50-cm coil length for C1 was selected since it gave the highest sensitivity.

#### 3.2.5. Sample Volume

The amount of standard sulfite injected into the system varied from 100 to 500 µL. As shown in Figure 4d, increasing the volume from 100 to 200 µL improves the sensitivity by 50%. However, for the 500-µL sample volume, there is at only a further increase of 10%. We therefore selected a 200-µL volume as the optimal sample volume.

### 3.3. Interference Study

Evaluation of some possible interference species was carried out in order to examine the selectivity of the developed Flow-GD-C4D system. The possible interference species was spiked at various concentration into 25 mg L^−1^ sulfite standard solution. The limit of tolerance is defined as the highest concentration of the species that gives the C4D signal not greater than ± 3 standard deviation (SD) of the mean of the C4D signal of the standard sulfite solution (SD). Table 1 summarizes the tolerance limit of possible interference species. Only the sugars and glycerol have a low limit of tolerance. Since samples of dried fruit extracts and fruit juices contain sugar, this problem was resolved by preparing the standard sulfite solutions with addition of sucrose at 2% (w/w) for the calibration plots when measuring sulfite in these samples (see Section 2.1).

### 3.4. Capability of the Proposed System for Turbid Samples

The normal flow injection systems employ an injection valve to introduce the sample with q reproducible volume. However, the sample solution must be clear to avoid clogging the injection valve. Thus, sample filtration or centrifugation are normally needed to obtain clear solutions, which is time-consuming. As discussed in Section 2.3 and Section 3.1, the flow System II shown in Figure 1b was suitable for the direct introduction of diluted turbid fruit juice without filtration. 

**Figure 5:**
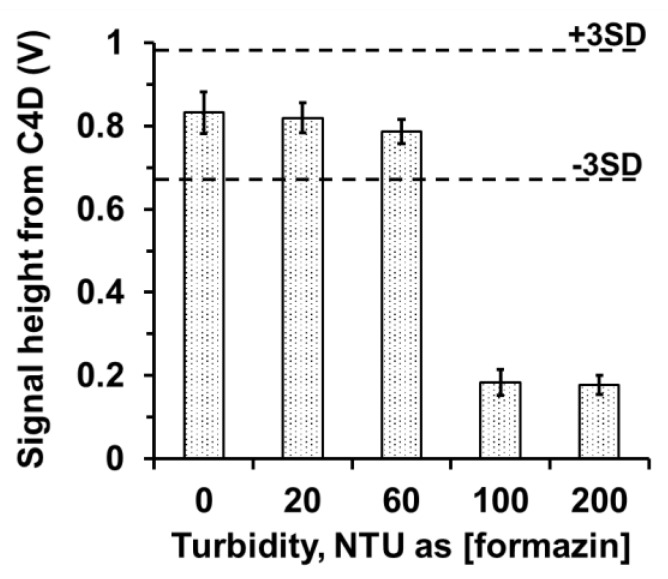
Tolerance limit of the developed system for turbidity on the standard solution of 25 mg L^−1^ SO_3_^2−^ (the tolerance limits of +/− 3SD).

**Figure A3:**
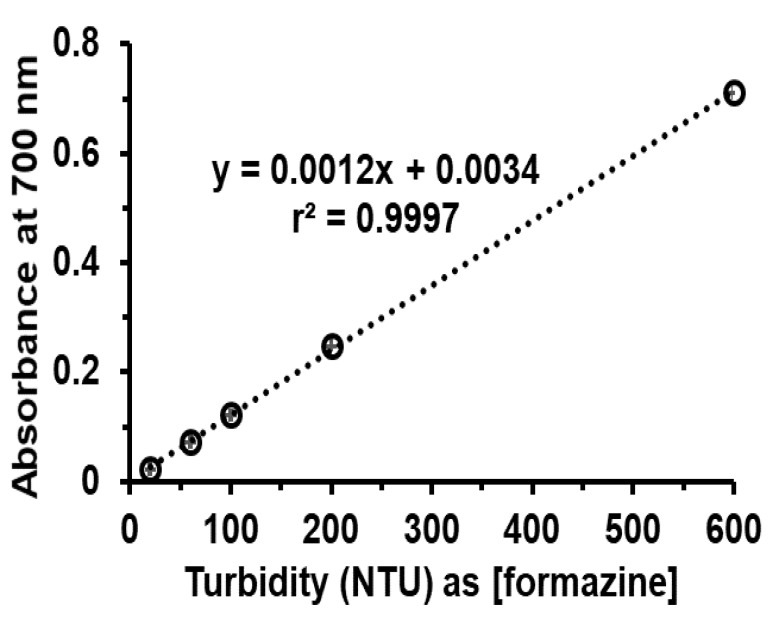
Calibration graph for turbidity in NTU using standard solutions of formazine.

However, colloidal matter in fruit juice sample may affect the diffusion process of SO_2_ through the PTFE membrane. We therefore investigated the tolerance limit of the system to turbidity. A standard 25-mg L^−1^ sulfite solution containing various concentrations of formazine (HACH, CO, USA), having turbidity ranging from 0 to 200 Nephelometric Turbidity Units (NTUs), was prepared. The tolerance limit is defined as the maximum turbidity giving a C4D signal of the standard sulfite solution < (mean − 3SD). As shown in Figure 5, the Flow-GD-C4D system can tolerate turbidity up to 60 NTUs. The turbidity of the samples of fruit juice after tenfold dilution with DI water is in the range of 20-50 NTUs (see Figure A3 and Table A2 in Appendix D in supplementary data). Thus, diluted fruit juice is suitable for direct introduction into the flow system.

### 3.5. Analytical Performances

Under the optimal condition described in Section 3.2, the analytical performance for the developed flow Systems I and II for the determination of total sulfite is tabulated in Table 2. Both systems provide a working range of 5 to 25 mg L^−1^ SO_3_^2−^ suitable for the sulfite analysis of most wines, extracts of dried fruit and fruit juices and also for the regulatory purposes, e.g., labeling required for sulfite content > 10 mg L^−1^ [7]. Good precision with < 2% RSD and the throughput of 24 injection h^−1^ were obtained. The limits of detection were 2.4 mg L^−1^ SO_3_^2−^ and 3.3 mg L^−1^ SO_3_^2−^ for flow Systems I and II, respectively (see footnote of Table 2 for definition of LOD). For the analysis of turbid samples, only the appropriate dilution of the samples is required.

There is a change of the baseline signal after each injection of either standard or sample solutions. However, the baseline returns to original value before the injection of the following standard or sample, indicating that the membrane is still in good condition (no clogging or tear of the membrane). During a measurement procedure, the performance of the PTFE membrane is checked by injecting a control solution of 10 mg L^−1^ SO_3_^2−^ after every six samples. The state of the PTFE membrane is based on whether the concentration of the control is within ±3SD of the back-calculated concentration using the calibration equation.

Table 3 lists the comparison of the characteristics of the various flow-based systems employing gas-diffusion separation and C4D detection of sulfite as reported in the literature. Most of the samples were clear wine. The limit of quantitation ranged from 0.1–7.8 mg L^−1^ SO_3_^2−^.

### 3.6. Application to Wine, Dried Fruit and Fruit Juice 

The developed flow System II was applied to the analysis of free and total sulfite in wine, dried fruits and fruit juices. To evaluate the accuracy of this method, the results for total sulfite were compared to those obtained from iodimetric titration [8], as shown in Table 4. The concentrations of total sulfite for the samples of white and red wines were comparable (paired *t*-test: *t*_stat_ = 1.98, *t*_crit_ = 2.36, *p* = 0.05), indicating that there were no systematic differences between the results of the two methods. However, it is observed (see Table 4) that total sulfite in dried fruits and juices obtained from iodimetric titration are always higher than our method. This is due to the presence of reducing species in fruit juice, such as ascorbic acid, citric acid and sugars, which can react with the iodine titrant. This would lead to the over estimation of total sulfite in these samples by iodimetric titration.

Table A3 (see Appendix E in Supplementary data) gives the free and total sulfite found in the samples. As expected, the concentrations of free sulfites are lower than total sulfite, which includes bound sulfites released upon the addition of NaOH. The developed method is therefore suitable for the analysis of both free and total sulfites.

## 4. Conclusions

This work presents a simple flow-based system incorporating an in-line gas-diffusion (GD) unit and a contactless conductivity detector for the analysis of total sulfite in wines, dried fruits and fruit juice. The analysis is based on the conversion of sulfite ions into SO_2_ gas via acidification with the generated gas diffusing through a PTFE membrane in a GD unit into a water acceptor stream. The dissolution of SO_2_ gas in the water acceptor gives ionic species that change the conductivity of the water plug, which is detected by the in-line C4D detector. Sample introduction is achieved through the aspiration of the sample for 12 s in order to avoid clogging problems with the injection valves. Wine and fruit juice are first diluted tenfold with DI water and then directly aspirated into the flow system. The PTFE membrane can tolerate a turbidity of samples up to 60 NTUs. The total sulfite content in samples were compared with iodimetric titration. The developed flow method is applicable to analysis of both clear and turbid samples.

## Figures and Tables

**Table 1:** Investigation of possible interfering species in wine for total sulfite analysis from triplicate injections of 25 mg L^−1^ SO_3_^2−^ standard solution.

Interfering Species (Unit)	Reported Level	Tolerance Limit for FIA-GD-C4D
Ethanol (%v/v)	16 ^a^	30
Sucrose (%w/w)	0.05–0.5 ^b^	0.05
Fructose (%w/w)	0.05–0.5 ^b^	0.05
Glucose (%w/w)	0.05–0.5 ^b^	0.01
Ascorbic acid (mg L^−1^)	1100–1200 ^b^	500
Tartaric acid (mg L^−1^)	1000–6,000 ^b^	1000
Citric acid (mg L^−1^)	Less than 1,000 ^b^	500
Glycerol (%w/v)	0.8–1 ^b^	0.5
CO_2_ (from HCO_3_^−^) (mg L^−1^)	Less than 2,000 ^b^	100

^a^ [27]; ^b^ [11].

**Table 2:** Analytical performance of the flow systems with an in-line GD-C4D system for the determination of total sulfite.

Analytical parameter	System I: FIA-GD-C4D	System II: Flow-C4D-G4D
1. Working range	5–25 mg L^−1^ SO_3_^2−^	5–25 mg L^−1^ SO_3_^2−^
2. Example of linear calibration, *r*^2^	*y* = [(8.73 ± 0.26) × 10^−2^]*x* – (0.20 ± 0.04), *r*^2^ = 0.997	*y* = [(8.48 ± 0.36) × 10^-2^]*x* – (0.12 ± 0.06), *r*^2^ = 0.995
3. Precision ^a^ (as RSD)	1.4 %	1.6 %
4. Limit of detection ^b^	2.4 mg L^−1^ SO_3_^2−^	3.3 mg L^−1^ SO_3_^2−^
5. Injection throughput	24 injections h^-1^	24 injections h^-1^
6. Type of samples	Wine	Wine, extracts of dried fruit, fruit juices
7. Sample pretreatment (see Section 2.2)	Addition of EDTA and NaOH Dilution with deionized water	Extraction with NaOH Dilution with deionized water

*x*: concentration of SO_3_^2−^ in unit of mg L^−1^, y: C4D signal height in volts. ^a^ nine replicates of 10 mg L^−1^ standard sulfite solution. ^b^ Limit of detection calculated from 5x(std. dev. of regression}/slope calibration).

**Table 3:** Comparison of a flow-based system coupled with a gas–liquid separation unit for the conductivity detection of total sulfite.

Method	Gas–Liquid Separation Unit/Type of Membrane	Acidification Solution	Acceptor Liquid	Sample(s)	Throughput (h^−1^)	Linear Range and Limit of Quantitation/Detection	Ref.
MPFS Conductometry	GD/Tubular PTFE	2 M H_3_PO_4_	1 mM H_2_O_2_	Wines (white, red and rose)	12	12.75-95.25 mg L^−1^ SO_3_^2−^ LOQ: 12.75 mg L^−1^ SO_3_^2−^	[11]
FIA Conductometry	GD/PTFE membrane	2 M HCl	Water	Wines (white, red and rose) and juices	120	1-50 mg L^−1^ SO_3_^2−^ LOQ: 0.10 mg L^−1^ SO_3_^2−^	[28]
FIA-SIA Contactless conductivity (C4D)	MBL-VP/-	1.5 M H_2_SO_4_	Water	Wines (white, and red)	26	10-200 mg L^−1^ SO_3_^2−^L LOQ: 0.30 mg L^−1^ SO_3_^2−^	[24]
FIA-SIA Contactless conductivity (C4D)	MBL-VP/-	1.5 M H_2_SO_4_	Water	White wines	24	10-200 mg L^−1^ SO_3_^2−^L LOQ: 7.68 mg L^−1^ SO_3_^2−^	[26]
FIA Contactless conductivity (C4D)	GD/PTFE membrane	1 M H_2_SO_4_	Water	Wines (white, and red)	24	5-25 mg L^−1^ SO_3_^2−^ LOD: 2.4 mg L^−1^ SO_3_^2−^	**This work**
				Dried fruits and juices	24	5-25 mg L^−1^ SO_3_^2−^ LOD: 3.3 mg L^−1^ SO_3_^2−^	

MPFS: multipumping flow system; FIA: flow injection analysis; SIA: sequential injection analysis; GD: gas-diffusion unit; MBL-VP: membraneless vaporization unit; PTFE: Polytetrafluoroethylene; C4D: capacitively coupled contactless conductivity detector; LOQ: limit of quantitation; LLOQ: lower limit of quantitation.

**Table 4:** Results of total sulfite content in wines, dried fruits and fruit juices determined by FIA-GD-C4D as compared to iodimetric titration.

Sample	Total Sulfite	
	This Work	Iodimetric Titration [8]
White wine #1 *	91.8 ± 1.2 ^a^	108.7 ± 8.4 ^a^
White wine #2 *	105.6 ± 0.4 ^a^	92.6 ± 8.8 ^a^
White wine #3 *	105.8 ± 1.6 ^a^	110.3 ± 2.3 ^a^
White wine #4 *	83.3 ± 0.8 ^a^	93.9 ± 0.9 ^a^
Red wine #1 *	101.2 ± 3.7 ^a^	112.8 ± 0.5 ^a^
Red wine #2 *	56.5 ± 2.1 ^a^	80.1 ± 1.7 ^a^
Red wine #3 *	78.8 ± 0.1 ^a^	79.7 ± 6.0 ^a^
Red wine #4 *	80.0 ± 0.1 ^a^	86.4 ± 8.8 ^a^
Dried guava	170.8 ± 0.7 ^b^	159.6 ± 4.0 ^b^
Dried mango	96.9 ± 0.8 ^b^	206.2 ± 2.3 ^b^
Apple juice #1	81.4 ± 0.3 ^a^	205.4 ± 2.4 ^a^
Apple juice #2	122.4 ± 1.6 ^a^	163.1 ± 0.5 ^a^
Lime juice	137.2 ± 1.8 ^a^	184.1 ± 8.8 ^a^

^a^ mg L^−1^ SO_3_^2−^; ^b^mg kg^−1^ SO_3_^2−^. * Paired *t*-test for white and red wines sample: *t*_stat_ = 1.98, *t*_crit_ = 2.36, *p* = 0.05.

## Appendix C. Back-Calculated Concentrations from Calibration Graphs Obtained from the Systems I and II

**Table A1:** Quantitative results of back-calculated concentrations from calibration graphs obtained from the Systems I and II.

Nominal Concentration	Back-Calculated Concentration (mg L^−1^ SO_3_^2−^)	
	System I	System II
5.0	5.16 ± 0.19	4.97 ± 0.28
10.0	9.96 ± 0.09	10.71 ± 0.51
15.0	13.53 ± 0.32	15.10 ± 0.63
20.0	20.86 ± 0.21	19.51 ± 0.80
25.0	24.40 ± 0.20	25.11 ± 0.24

Paired *t*-test: *t*_stat_ (0.68) < *t*_crit_ (3.16); *p* = 0.05.

## Appendix D. Investigation of Tolerance Limit for Turbidity Suitable for Diluted Cloudy Juice Samples

**Table A2:** Measured turbidity values in NTU of cloudy fruit juice samples from the calibration graph in Figure A2.

Sample	Turbidity, NTU (n = 3)	
	Tenfold Diluted Sample	Original Sample
Apple juice #1	19.5 ± 0.4	194.7 ± 4.2
Apple juice #2	19.3 ± 0.2	193.1 ± 1.7
Lime juice	50.1 ± 0.5	501.4 ± 5.4

## Appendix E. Results of the Determination of Free and Total Sulfite

**Table A3:** Results of the determination of free and total sulfite in wine, dried fruit, and fruit juice samples obtained using the flow Systems I and II.

Sample	Free sulfite (mg L^−1^ SO_3_^2−^)	Total sulfite (mg L^−1^ SO_3_^2−^)
White wine #1	67.4 ± 2.2	183.5 ± 1.1
White wine #2	78.9 ± 5.4	211.6 ± 1.6
Red wine #1	88.6 ± 1.2	113.0 ± 2.1
Red wine #2	102.6 ± 1.6	137.7 ± 6.4
Dried fruit	n.d.	341.7 ± 6.5
Juice	107.8 ± 3.5	244.8 ± 3.2

n.d.: not detected.

## References

[1] Iammarino M., Ientile A.R., Di Taranto A. (2017). Sulphur dioxide in meat products: 3-year control results of an accredited Italian laboratory. Food Addit. Contam. Part B.

[2] Taylor S.L., Higley N.A., Bush R.K., Chichester C.O., Mrak E.M., Schweigert B.S. (1986). Sulfites in Foods: Uses, Analytical Methods, Residues, Fate, Exposure Assessment, Metabolism, Toxicity, and Hypersensitivity. Advances in Food Research.

[3] Brody T.O.M., Brody T.O.M. (1999). 10—Inorganic nutrients. Nutritional Biochemistry.

[4] Vally H., Misso N.L.A., Madan V. (2009). Clinical effects of sulphite additives. Clin. Exp. Allergy.

[5] Gunnison A.F., Jacobsen D.W., Schwartz H.J. (1987). Sulfite Hypersensitivity. A Critical Review. Crc Crit. Rev. Toxicol..

[6] WHO (2002). Safety Evaluation of Certain Food Additives and Contaminants/Prepared by the Fifty-Seventh Meeting of the Joint FAO/WHO Expert Committee on Food Additives (JECFA).

[7] Timbo B., Koehler K.M., Wolyniak C., Klontz K.C. (2004). Sulfites—A Food and Drug Administration Review of Recalls and Reported Adverse Events. J. Food Prot..

[8] Vahl J.M., Converse J.E. (1980). Ripper procedure for determining sulfur dioxide in wine: Collaborative study. J. Assoc. Off. Anal. Chem..

[9] De Paula N.T.G., Barbosa E.M.O., da Silva P.A.B., de Souza G.C.S., Nascimento V.B., Lavorante A.F. (2016). In-line electrochemical reagent generation coupled to a flow injection biamperometric system for the determination of sulfite in beverage samples. Food Chem..

[10] Total Sulfite Analyzer | Labcompare.com. https://www.labcompare.com/6184-Total-Sulfite-Analyzer/56827-Total-Sulfite-and-SO2-H2S-Analyzer/?pda=6184|56827_2_0|||#productdetails.

[11] Danchana K., Clavijo S., Cerda V. (2019). Conductometric Determination of Sulfur Dioxide in Wine Using a Multipumping System Coupled to a Gas-Diffusion cell. Anal. Lett..

[12] Henriquez C., Horstkotte B., Cerda V. (2014). A highly reproducible solenoid micropump system for the analysis of total inorganic carbon and ammonium using gas-diffusion with conductimetric detection. Talanta.

[13] Gimenez-Gomez P., Gutierrez-Capitan M., Puig-Pujol A., Capdevila F., Munoz S., Tobena A., Miro A., Jimenez-Jorquera C. (2017). Analysis of free and total sulfur dioxide in wine by using a gas-diffusion analytical system with pH detection. Food Chem..

[14] Bartroli J., Escalada M., Jorquera C.J., Alonso J. (1991). Determination of Total and Free Sulfur Dioxide in Wine by Flow Injection Analysis and Gas-diffusion using p-aminoazobenzene as the Colorimetric Reagent. Anal. Chem..

[15] Santos J.C.C., Korn M. (2006). Exploiting Sulphide Generation and Gas Diffusion Separation in a Flow System for Indirect Sulphite Determination in Wines and Fruit Juices. Microchim. Acta.

[16] Silva C.R., Gomes T.F., Barros V.A., Zagatto E.A. (2013). A multi-purpose flow manifold for the spectrophotometric determination of sulphide, sulphite and ethanol involving gas diffusion: Application to wine and molasses analysis. Talanta.

[17] Lin J., Hobo T. (1996). Flow-injection analysis with chemiluminescent detection of sulphite using Na_2_CO_3_-NaHCO_3_-Cu^2+^ system. Anal. Chim. Acta.

[18] Araujo A.N., Couto C.M.C.M., Lima J.F.C.L., Montenegro M.C.B.S.M. (1998). Determination of SO_2_ in Wines Using a Flow Injection Analysis System with Potentiometric Detection. J. Agric. Food Chem..

[19] Gonçalves L.M., Grosso Pacheco J., Jorge Magalhães P., António Rodrigues J., Araújo Barros A. (2010). Determination of free and total sulfites in wine using an automatic flow injection analysis system with voltammetric detection. Food Addit. Contam. Part A.

[20] Chinvongamorn C., Pinwattana K., Praphairaksit N., Imato T., Chailapakul O. (2008). Amperometric Determination of Sulfite by Gas Diffusion-Sequential Injection with Boron-Doped Diamond Electrode. Sensors.

[21] Martins P.R., Popolim W.D., Nagato L.A.F., Takemoto E., Araki K., Toma H.E., Angnes L., Penteado M.D.V.C. (2011). Fast and reliable analyses of sulphite in fruit juices using a supramolecular amperometric detector encompassing in flow gas diffusion unit. Food Chem..

[22] Ratanawimarnwong N., Pluangklang T., Chysiri T., Nacapricha D. (2013). New membraneless vaporization unit coupled with flow systems for analysis of ethanol. Anal. Chim. Acta.

[23] Alahmad W., Pluangklang T., Mantim T., Cerda V., Wilairat P., Ratanawimarnwong N., Nacapricha D. (2018). Development of flow systems incorporating membraneless vaporization units and flow-through contactless conductivity detector for determination of dissolved ammonium and sulfide in canal water. Talanta.

[24] Chantipmanee N., Alahmad W., Sonsa-ard T., Uraisin K., Ratanawimarnwong N., Mantim T., Nacapricha D. (2017). Green analytical flow method for the determination of total sulfite in wine using membraneless gas–liquid separation with contactless conductivity detection. Anal. Methods.

[25] Lin J., Zhu Y., Cheng W., Wang J., Wu B., Wang J. (2014). Determination of Free and Total Sulfite in Red Globe Grape by Ion Chromatography. Food Sci. Technol. Res..

[26] Kraikaew P., Pluangklang T., Ratanawimarnwong N., Uraisin K., Wilairat P., Mantim T., Nacapricha D. (2019). Simultaneous determination of ethanol and total sulfite in white wine using on-line cone reservoirs membraneless gas-liquid separation flow system. Microchem. J..

[27] Varela C., Dry P.R., Kutyna D.R., Francis I.L., Henschke P.A., Curtin C.D., Chambers P.J. (2015). Strategies for reducing alcohol concentration in wine. Aust. J. Grape Wine Res..

[28] Araujo C., Decarvalho J., Mota D., Dearaujo C., Coelho N. (2005). Determination of sulphite and acetic acid in foods by gas permeation flow injection analysis. Food Chem..

